# Pneumothorax

**Published:** 1904-08-13

**Authors:** 


### Hospital Clinics.

### PNEUMOTHORAX.

Regarded apart from clinical experience pneumothorax would inevitably be considered as a condition having a peculiarly distinct and definite onset, and attended by acute manifestations which could not fail to impress themselves upon the attention of the patient. A rush of air into the pleural cavity, which, in the nature of things, can hardly be other than sudden, and which involves a prompt compression and folding-up of the lung, often with extreme mechanical displacement of the heart, and a complete alteration of the pressure conditions within the thorax, is an event which, on theoretical grounds, would be naturally expected to produce an acute and unmistakable subjective experience. That, in certain cases, this is so, is beyond question. But it is not an invariable or even the more usual order of events. Indeed, it is hardly too much to say that, in the majority of cases, pneumothorax develops without any recognition on the part of the patient and often also escapes detection by the physician. This general proposition finds full illustration in the report recently issued by the Pathological Department of the Brompton Hospital,[1] which includes a record of the post-mortem examinations made in the department during the three years ending April 1903. Amongst these are accounts of autopsies on 19 cases of tuberculous pneumothorax, and in no less than eight of these the personal history was free from any acute event suggesting the date of origin of the pneumothorax. Further, there were in these cases few or no symptoms indicating the existence of such a condition, and the discovery was either made during the physical examination or at the autopsy on opening the chest. This record is in entire harmony with the experience of all physicians who have had considerable practice in diseases of the chest. The explanation of this absence of symptoms which on *a priori* grounds might have been expected to attend a condition of pneumothorax is in some cases a fairly obvious one. Prior to the occurrence of the pneumothorax, more or less extensive adhesions have been formed between the parietal and visceral layers of the pleura and thus when, by rupture of the latter, air is admitted into the pleural cavity the extent of the inrush is limited and confined, and corresponding to this the compression of the lung is only partial. This is illustrated in four out of the eight cases above alluded to, and it is a frequent occurrence in pneumothorax developing in the course of pulmonary phthisis. But it is not true in all cases, as is shown in the report to which allusion has just been made. For in no less than four of the eight cases of pneumothorax existing without symptoms the pneumothorax was found to be either "total" or "practically total." In all these instances it is to be observed that the affected lung was the subject of chronic pulmonary tuberculosis or of acute caseous disease with rapid softening, and thus it may be presumed that a large part of the lung was out of action prior to the occurrence of the pneumothorax, and that it contributed therefore but a small share to the success of the thoracic respiration. Hence the compression of the lung in these circumstances would not inflict any special embarrassment on the respiratory efficiency, and anything like sudden dyspnœa as an index of the supervention of the pneumothorax would fail to occur. Again, it must be remembered that when pneumothorax develops in advanced phthisis pulmonalis the patient will probably at the time be resting, and perhaps in the recumbent posture, so that the respiratory demand and effort are reduced to a low level. In any circumstances, the escape of air into the pleural cavity can hardly be a gradual process, and therefore the explanation which applies to the absence of symptoms in cases of slow effusion of fluid into the pleural cannot be adopted in reference to pneumothorax. But recognising this, and giving full weight to the considerations already advanced it still remains somewhat surprising that pneumothorax should so frequently occur without evidences that force themselves on the attention of the patient. This applies, perhaps, with special force to the condition when it affects the left side and produces, as it then does, considerable cardiac displacement. The moral, of course, is that the physician who takes charge of cases of pulmonary tubercle must not be content to rest on the subjective sensations of his patients but must satisfy himself of the actual state of matters by frequent and thorough physical examination.

Another aspect of pneumothorax, not quite free from obscurity, is provided by cases in which there is a rush of air into the pleural cavity without any previous history or existing symptom of disease, either in the chest or elsewhere. Doubtless in every case of this order it is wise, at least for a time, to regard the patient as more or less definitely under suspicion in reference to the existence of early pulmonary tubercle, for it is precisely in early tubercle that pneumothorax, if it occurs, is likely to be complete and free from the limiting influence of pleural adhesions. By evil chance, as it were, an early tubercular focus forms close to the visceral pleura, and when perforation of the latter occurs the air has access to a pleural cavity which has not been modified by a more or less chronic tubercular process. Hence an extreme pneumothorax may occur in a patient who previously has given very slight evidences—and possibly none at all—of pulmonary disease. The principle of this is illustrated in one of the cases in the Brompton Report, in which, with old and almost stationary disease in the upper part of the lung, the patient had developed a pneumothorax due to a small patch of caseation near the surface of a relatively healthy lower lobe. Hence it cannot be questioned that the safe position in every case of pneumothorax arising without obvious explanation is to consider the case as possibly one of early pulmonary tubercle. There are, however, a sufficient number of instances to show that all such cases are not tuberculous, and others which, in all probability are in the same category. Some of these abstracted from recent records may be usefully noted. B. Stiller[2] relates the case of a young man who, after a violent sneeze, was seized with extreme abdominal pain, and showed the physical signs of right pneumothorax, including considerable displacement of the heart and liver. There was amphoric breathing all over the right chest and this, together with the marked displacement of the organs just named, suggests a valvular opening in the visceral pleura permitting more air to enter with each inspiration but preventing the egress of air on expiration. Morphine relieved the pain, and in the course of a week all signs of pneumothorax had disappeared; there were at no time any evidences of pulmonary disease. Fussell and Reisman[3] record a similar case in which the pneumothorax followed an attack of coughing, the patient, a man of 71 years, being quite well three months after the attack. The same authors report a case in which pneumothorax occurred in a young woman during sleep, and seven years later the patient had remained in good health and entirely free from all evidences of pulmonary disease. In 1902 two patients were shown at the Polyclinic[4] as examples of pneumothorax without any sign of tubercular or other disease of the lung. The one patient was a man of 45 years and the other a man of 60, and in each instance the pneumothorax had occurred while the patient was resting in bed. There are other examples in which the pneumothorax follows an injury. In some of these there may of course be fracture of a rib, with, as a result, tearing of the visceral pleura, even though no crepitus can be detected. This is suggested in an instance related by Morris,[5] in which the pneumothorax followed a fall and was accompanied by subcutaneous emphysema. No evidences of a fractured rib could be detected, and the patient regained full expansion of the lung in the course of 14 days. A parallel experience in a boy, after being run over by a cab, has recently been reported by Stanley Taylor.[6] In other instances there can be no possible suggestion of a fractured rib, as in a case reported by Bevan,[7] in which pneumothorax occurred in a clerk after carrying a pile of ledgers. Here also the patient is reported as perfectly well and able to return to work in the course of a fortnight. Of course in cases where there has been a violent attack of sneezing or coughing, and the same is true of a strain, as in lifting heavy weights, it is less difficult to understand the production of pneumothorax than it is when the condition occurs during rest or sleep. Such acts involve, in consequence of closure of the glottis on the one hand, and muscular strain on the walls of the thorax on the other, considerable increase in the intra-pulmonary pressure, during which it is conceivable some of the air-cells may be ruptured. But even then there remains the difficulty of rupture of the pulmonary pleura. One possibility in this direction is worth considering. If at any time a patient has suffered from an attack of pleurisy, there may well be one or more adhesions, limiting, perhaps over a very narrow district, the free play of the lung. In such circumstances a localised emphysema is a common development, and as this is apt to affect the superficial part of the lung, it may readily be the case that a sudden increase of intra-pulmonary pressure may rupture both the dilated air-vesicles and the thinned portion of the visceral pleura that covers them. To prove this in any individual case, at least during life, is difficult or impossible, but it may well be that in such a suggestion an explanation of the development of pneumothorax in otherwise healthy patients may be found. It is of interest in this connection to recall the fact that at one time it was believed that air might be "secreted" by the pleura, and might in this way accumulate in the pleural cavity. This was fully believed in by Graves,[8] who records several cases in support of this theory. But "pneumothorax from gaseous secretion" will hardly be accepted as a justifiable diagnosis at the present day. The argument as stated against the view adopted by Graves may be found in Wilson Fox's classical treatise on diseases of the chest. Regarding the prognosis and treatment of cases of so-called "simple pneumothorax," a general review of the recorded cases carries the distinct impression that there is little or no claim for heroic measures. In the great majority of instances nothing was done beyond relief of the pain and cough by morphine, with subsequent rest in bed, and perhaps strapping the affected side of the chest. It can but rarely be necessary to remove the accumulated air from the chest. In occasional instances, when the pressure becomes very great in consequence, perhaps, of the existence of a valvular opening in the pulmonary pleura, this may, however, be demanded; and, if so, it should be accomplished by a puncture with a hollow needle, thus permitting the excess of air to escape. But aspiration is always inadvisable. To extract the air by this method is to run the risk of reducing the pressure within the pleural cavity below the intra-pulmonary pressure, with a probable reopening of the wound on the surface of the lung and a further aggravation of the pneumothorax. A good plan is that advised by Béclère,[9] the fine trocar or needle used to puncture the chest having attached to it an indiarubber tube terminating in a glass tube bent to a right angle and the free end of which is kept under water. In this way the entrance of air, with possible septic matter, from the outside into the chest is avoided, and the passage of bubbles of gas through the water is an index of the escape of air under pressure from the pneumothorax. It is, however, only in cases where extreme or increasing disturbance from intra-thoracic pressure exists that puncture is indicated. Altogether, the immediate outlook in cases of pneumothorax is less serious than might be expected. Even in cases of tubercle it does not, in the majority of instances, seem to hasten a fatal issue. And in some cases, perhaps by reducing to quiescence a lung which is the seat of active disease, it even appears to have an influence favourable rather than otherwise.

[1] Report of the Pathological Department of the Brompton Hospital, 1900–03. [2] Brit. Med. Jour., Epitome, July 20, 1901. [3] Ibid., Dec. 27, 1902. [4] The Polyclinic Jour., 1902. [5] Lancet, Oct. 25, 1902. [6] Ibid., July 30, 1904. [7] Ibid., June 11, 1904. [8] Graves's Clinical Medicine. [9] Med. Review., Vol. III., p. 340.

